# The value of complete blood count for the prognosis analysis of preoperative esophageal squamous cell carcinoma

**DOI:** 10.1186/s12885-021-08789-2

**Published:** 2021-09-30

**Authors:** Xiang Lv, Songtao Han, Bin Xu, Yuqin Deng, Yangchun Feng

**Affiliations:** 1 Clinical Laboratory Center, Cancer Hospital Affiliated to Xinjiang Medical University, Xinjiang, China; 2 Clinical Laboratory Center, Hospital of Traditional Chinese Medicine affiliated to Xinjiang Medical University, Xinjiang, China; 3 West China hospital affiliated to Sichuan University, Sichuan, China

## Abstract

**Objective:**

To investigate the predictive value of preoperative complete blood count for the survival of patients with esophageal squamous cell carcinoma.

**Methods:**

A total of 1587 patients with pathologically confirmed esophageal squamous cell carcinoma who underwent esophagectomy in the Cancer Hospital Affiliated to Xinjiang Medical University from January 2010 to December 2019 were collected by retrospective study. A total of 359 patients were as the validation cohort from January 2015 to December 2016, and the remaining 1228 patients were as the training cohort. The relevant clinical data were collected by the medical record system, and the patients were followed up by the hospital medical record follow-up system. The follow-up outcome was patient death. The survival time of all patients was obtained. The Cox proportional hazards regression model and nomogram were established to predict the survival prognosis of esophageal squamous cell carcinoma by the index, their cut-off values obtained the training cohort by the ROC curve. The Kaplan-Meier survival curve was established to express the overall survival rate. The 3-year and 5-year calibration curves and C-index were used to determine the accuracy and discrimination of the prognostic model. The decision curve analysis was used to predict the potential of clinical application. Finally, the validation cohort was used to verify the results of the training cohort.

**Results:**

The cut-off values of NLR, NMR, LMR, RDW and PDW in complete blood count of the training cohort were 3.29, 12.77, 2.95, 15.05 and 13.65%, respectively. All indicators were divided into high and low groups according to cut-off values. Univariate Cox regression analysis model showed that age (≥ 60), NLR (≥3.29), LMR (< 2.95), RDW (≥15.05%) and PDW (≥13.65%) were risk factors for the prognosis of esophageal squamous cell carcinoma; multivariate Cox regression analysis model showed that age (≥ 60), NLR (≥3.29) and LMR (< 2.95) were independent risk factors for esophageal squamous cell carcinoma. Kaplan-Meier curve indicated that age <  60, NLR < 3.52 and LMR ≥ 2.95 groups had higher overall survival (*p* <  0.05). The 3-year calibration curve indicated that its predictive probability overestimate the actual probability. 5-year calibration curve indicated that its predictive probability was consistent with the actual probability. 5 c-index was 0.730 and 0.737, respectively, indicating that the prognostic model had high accuracy and discrimination. The decision curve analysis indicated good potential for clinical application. The validation cohort also proved the validity of the prognostic model.

**Conclusion:**

NLR and LMR results in complete blood count results can be used to predict the survival prognosis of patients with preoperative esophageal squamous cell carcinoma.

## Background

Malignancy is a major public health problem in the world, according to the 2019 global cancer data report, esophageal cancer incidence and mortality rank the 7th and 6th in all cancer, respectively [1]. Esophageal cancer could be divided into two categories according to histological types, including esophageal squamous cell carcinoma (ESCC) and esophageal adenocarcinoma (EAC). Esophageal squamous cell carcinoma mainly distributed in Asia, accounting for about 90%; esophageal adenocarcinoma mainly distributed in Europe, accounting for about 70–80% [2, 3]. The middle-aged and elderly had high incidence of esophageal cancer, the early clinical manifestations are mainly dysphagia, burning sensation under the sword, weight loss and other atypical symptoms, the later could be manifested as progressive dysphagia, so the early symptoms are easy to miss the diagnosis, most of the diagnosis is advanced, the five-year survival rate is only 10–20% [4, 5], early and mid-term esophageal cancer should be treated by surgery, but the overall survival rate has not changed significantly, so how to evaluate the prognosis of patients before surgery has important clinical significance for clinicians and patients, which could help clinicians to change the treatment plan, while improving the survival confidence of patients.

Over the past few decades, it has been confirmed that depth of invasion, lymph node metastasis, TNM stage, and various other factors [6] were important prognosis factors of esophageal cancer, meanwhile, more and more experiments in recent years have demonstrated that inflammation is associated with the survival of malignant tumors. Complete blood count (CBC) is one of the most common clinical laboratory tests, and absolute counts of neutrophils, lymphocytes, and monocytes reflect the inflammatory response and the overall immune status of the body. Peripheral blood prognostic inflammatory markers include neutrophil to lymphocyte ratio (NLR), neutrophil to monocyte ratio (NMR), lymphocyte to monocyte ratio (LMR), red blood cell distribution width (RDW) and blood cell distribution width (PDW), which have been demonstrated to be closely related to the prognosis of various cancers [6, 7]. In clinical laboratories, because blood routine tests are mandatory for patients and cheap, experimental data are relatively easy to obtain. Therefore, it is of great clinical significance to predict the prognosis of patients with esophageal squamous cell carcinoma by the results of blood routine test items.

## Materials and methods

### Patient

This retrospective study was approved by the Ethics Committee of Cancer Hospital Affiliated to Xinjiang Medical University. All cases of esophageal cancer were diagnosed by pathological results. A total of 1587 cases of esophageal squamous cell carcinoma were collected, who underwent esophagectomy and were diagnosed in the Cancer Hospital Affiliated to Xinjiang Medical University from January 2010 to December 2019. In them, 359 cases from January 2015 to December 2016 were selected as the validation cohort, and the remaining 1228 cases were as the training cohort. All patients with esophageal squamous cell carcinoma were pathologically staged by experienced oncologists according to the 8th edition of TNM staging issued by the American Joint Committee on Cancer (AJCC). Inclusion criteria:1) histopathologically confirmed ESCC without distant metastasis (TNM-I-III) from January 2010 to December 2019 were included in the study; 2) at least 6 lymph nodes were examined for pathological diagnosis; 3) no chemotherapy and/or radiotherapy before and after surgery; 4) preoperative CBC was obtained 1 time before esophagectomy. Exclusion criteria: 1) previous or concomitant other cancer; 2) serious complications or death within 30 days after surgery; 3) preoperative systemic inflammatory response syndrome (SIRS) or evidence of infection, autoimmune disease. The last follow-up date was 21 February 2021.

### Statistical analysis

The area under the curve (AUC) was calculated by receiver operating characteristic curve (ROC) and the optimal cutoff value for continuous variables was calculated. The correlation between each indicator and pathological parameters was indicated by spearman correlation coefficient, univariate and multivariate analyses were performed using the Cox regression analysis model to assess the effect of multiple covariates on survival outcomes; nomograms were generated with Cox regression coefficients, Kaplan-Meier survival curves were established to express overall survival, their accuracy and discrimination were indicated using the 3- and 5-year calibration curves and c-index, their potential for clinical application was indicated using the decision curve analyses, and finally validated using the validation cohort. For all analyses, *p* <  0.05 was defined as significant. Statistical analysis was performed using SPSS version 26.0 (SPSS, Chicago, IL) and the rms, survival, survivalROC, and rmda packages of R language.

## Results

### Cut-off value of every test index

**Fig. 1:**
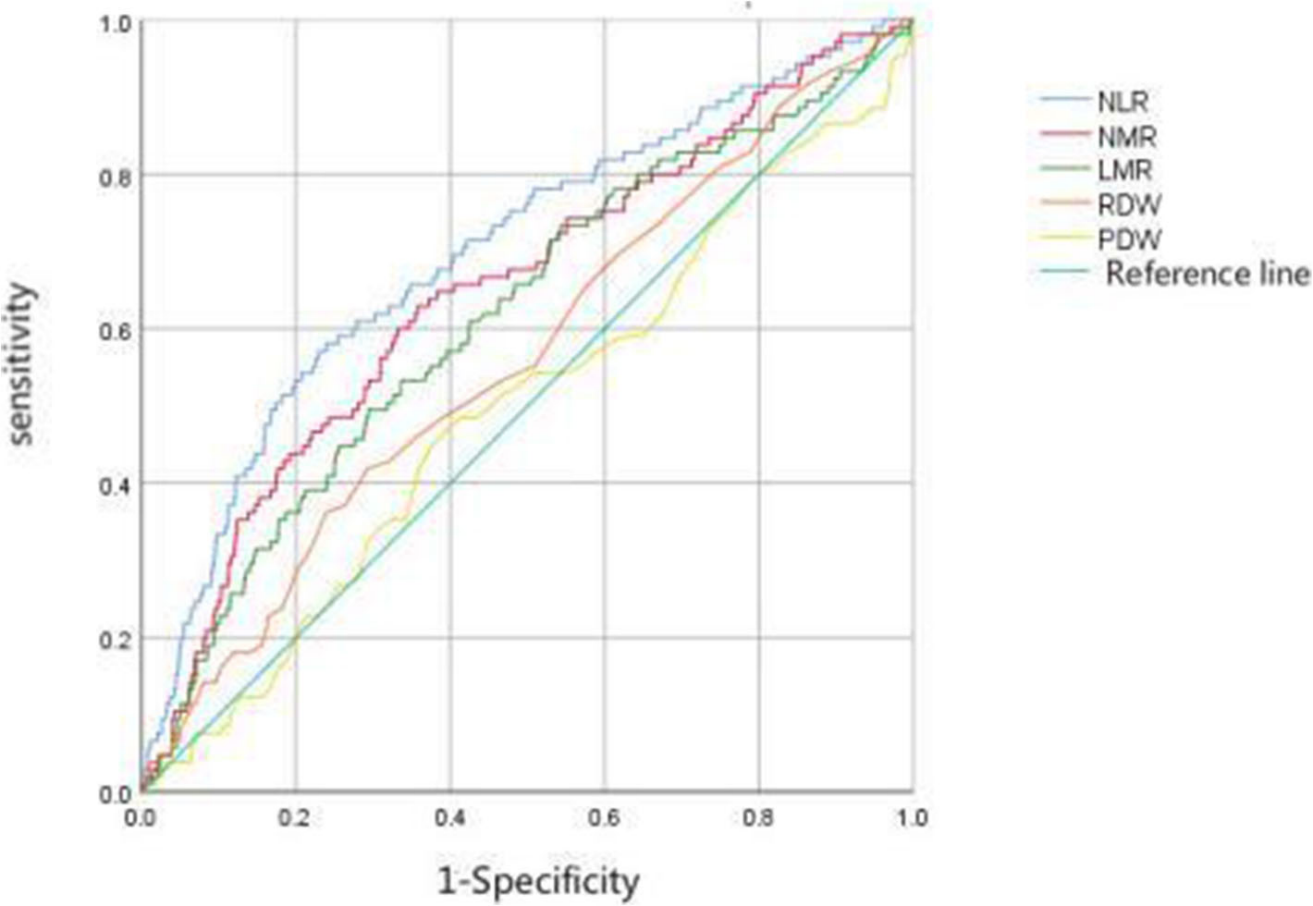
ROC curve of CBC index based on training cohort. NOTE: NLR:neutrophil to lymphocyte ratio;NMR:neutrophil to monocyte ratio;LMR:lymphocyte to monocyte ratio;RDW: red blood cell distribution width;PDW: blood cell distribution width

Taking whether the patient died in the final result as the outcome event, ROC curve was used to find out whether there was an appropriate cut-off value for every test items to predict the patient’s death outcome, and then the significance of every indicators in prognosis was demonstrated. The cut-off values of relevant quantitative NLR, NMR, LMR, RDW, PDW and other indicators were 3.29, 12.77, 2.95, 15.05 and 13.65%,respectively (Table 1, Fig. 1).

**Table 1:** ROC Analysis Results of Significant Quantitative

Index	Cut-off value	AUC	P
NLR	3.29	0.615	<0.001
NMR	12.77	0.516	<0.001
LMR	2.95	0.598	<0.001
PDW	15.05	0.489	0.275
RDW	13.65	0.498	0.179

NOTE:NLR:neutrophil to lymphocyte ratio;NMR:neutrophil to monocyte ratio;LMR:lymphocyte to monocyte ratio;RDW: red blood cell distribution width;PDW: blood cell distribution width

### Clinicopathological features of patients

The clinicopathological characteristics of the patients in the training cohort (*n* = 1228) and validation cohort (*n* = 359) in Table 2. The male to female ratio was 2.21:1 and 2.55:1 in the training and validation cohorts, respectively. In the training cohort Age > 60 (66.2%) and tumor location in the middle (72.6%) was significantly higher than < 60 (33.8%) and tumor location in the upper and lower segments (27.4%). In the pathological stage, T3 (64.6%) was the most depth of tumor invasion group, which was related to the diagnosis of advanced ESCC patients. 40.9% of ESCC patients had no lymph node metastasis. In Pathological staging, stage II (41.2%) and III (50.8%) patients accounted for the vast majority.

**Table 2:** Clinicopathological features and CBC index of esophageal squamous cell carcinoma

characteristic	Training cohort(n = 1228)		Validation cohort(n = 359)	
	Number of patients	Composition ratio (%)	Number of patients	Composition ratio (%)
Sex				
Male	845	68.8%	258	71.9%
Female	383	31.2%	101	28.1%
Age				
< 60 years	415	33.8%	111	30.9%
≥ 60 years	813	66.2%	248	69.1%
NLR				
< 3.29	864	70.4%	252	70.2%%
≥ 3.29	364	29.6%	107	29.8%%
NMR				
< 12.77	1013	82.5%	289	80.5%
≥ 12.77	215	17.5%	70	19.5%
LMR				
< 2.95	421	34.3%	126	35.1%
≥ 2.95	807	65.7%	233	64.9%
RDW				
< 15.05%	786	64%	234	65.2%
≥ 15.05%	442	36%	125	34.8%
PDW				
< 13.65%	1103	89.8%	324	90.3%
≥ 13.65%	125	10.2%	35	9.7%
Tumor location				
Upper	49	4.0%	12	3.3%
Middle	892	72.6%	258	71.9%
Lower	287	23.4%	89	24.8%
Depth of tumor invasion				
pT1	106	8.6%	27	7.5%
pT2	301	24.5%	87	24.2%
pT3	793	64.6%	230	64.1%
pT4	28	2.3%	15	4.2%
Lymph node metastasis				
pN0	502	40.9%	149	41.5%
pN1	502	40.9%	134	37.3%
pN2	224	18.2%	76	21.2%
Differentiation				
G1	61	5%	15	4.2%
G2	693	56.4%	210	58.5%
G3	474	38.6%	134	37.3%
Stage				
I	98	8%	27	7.5%
II	506	41.2%	141	39.3%
III	624	50.8%	191	53.2%

NOTE:NLR:neutrophil to lymphocyte ratio;NMR:neutrophil to monocyte ratio;LMR:lymphocyte to monocyte ratio;RDW: red blood cell distribution width;PDW: blood cell distribution width;HR: hazard ratio; CI: confidence interval

Based on the correlation between various indicators in the training cohort and pathological parameters (Table 3), It could be concluded that NLR, NMR and PDW were significantly correlated with Depth of invasion, Lymph node metastasis and Stage (*p* <  0.05), LMR is correlated with Lymph node metastasis, Differentiation and Stage (p <  0.05), and RDW is correlated with Stage (p <  0.05).

**Table 3:** Correlation between clinicopathological parameters and CBC in esophageal squamous cell carcinoma

Variables	Cases	NLR		R	P	LMR		R	P	NMR		R	P	RDW		R	P	PDW		R	P
		<3.29	≥3.29			<2.95	≥2.95			<12.77	≥12.77			<13.65	≥13.65			<15.05	≥15.05		
Gender				0.063	0.026			0.021	0.460			0.041	0.147			0.015	0.595			0.125	<0.001
Male	845	578	267			284	561			706	139			545	300			495	350		
Female	383	286	97			137	246			307	76			241	142			220	263		
Age (years)				0.004	0.894			0.028	0.326			0.008	0.792			0.026	0.354			0.022	0.436
< 60	415	293	122			150	265			344	71			273	142			248	167		
≥60	813	571	242			271	542			669	144			513	300			467	346		
Tumor location				0.047	0.102			0.042	0.140			0.022	0.451			0.017	0.546			0.027	0.346
Upper/Middle	941	651	290			333	608			772	169			598	343			541	400		
Lower	287	213	74			88	199			241	46			188	99			174	113		
Depth of invasion				0.084	0.033			0.054	0.314			0.123	<0.001			0.077	0.063			0.098	0.008
pT1	106	85	21			30	76			95	11			80	26			78	28		
pT2	301	221	80			104	197			267	34			184	117			169	132		
pT3	793	540	253			274	519			631	162			505	288			454	339		
pT4	28	18	10			13	15			20	8			17	11			14	14		
Lymph node metastasis				0.707	<0.001			0.420	0.033			0.123	<0.001			0.052	0.193			0.056	0.041
pN0	502	401	101			160	342			441	61			336	166			306	196		
pN1	502	337	165			180	322			389	113			309	193			282	220		
pN2	224	126	98			81	143			183	41			141	83			127	97		
Differentiation				0.002	0.998			0.626	<0.001			0.027	0.646			0.024	0.697			0.029	0.592
G1	61	43	18			20	41			52	9			36	25			32	29		
G2	693	488	205			216	477			566	127			444	249			409	284		
G3	474	333	141			185	289			395	79			306	168			274	200		
Stage				0.183	<0.001			0.167	0.041			0.118	<0.001			0.077	0.027			0.548	<0.001
I	98	80	18			23	75			89	9			75	23			72	26		
II	506	397	109			176	330			436	70			319	187			287	219		
III	624	387	237			222	402			488	136			392	232			356	268		

NOTE:NLR:neutrophil to lymphocyte ratio;NMR:neutrophil to monocyte ratio;LMR:lymphocyte to monocyte ratio;RDW: red blood cell distribution width;PDW: blood cell distribution width

### Univariate and multivariate cox regression analysis of various indicators

Univariate Cox regression analysis was used to calculate the value of gender, age, every classification index, tumor location, depth of invasion, lymph node metastasis, differentiation and pathological stage for the prognosis of esophageal cancer, respectively, and it could be found (Table 4) that age (*p* <  0.001), NLR (*p* <  0.001), LMR (p <  0.001), RDW (*p* = 0.049), PDW (*p* = 0.016), depth of invasion (*p* <  0.001), lymph node metastasis (p <  0.001) and stage (p <  0.001) were prognostic factors for esophageal cancer; multivariate Cox regression analysis could reveal that age (p <  0.001), NLR (p <  0.001), LMR (*p* = 0.001), depth of invasion (p <  0.001), lymph node metastasis (*p* = 0.016) and stage (*p* <  0.036) were independent risk factors for the prognosis of esophageal cancer. The age ≥ 60 years group showed a 1.341-fold (1.169–1.538) increased risk of death than the < 60 years group;NLR ≥ 3.52 group vs. < 3.52 group, with a 1.578-fold (1.373–1.814) increased risk of death. The LMR < 2.95 group was associated with a 1.265-fold (1.107–1.445) increased risk of death compared with the LMR ≥ 2.95 group.

**Table 4:** Univariate and multivariate Cox proportional hazards regression models in patients with ESCC

	Univariate analysis HR (95% CI)	*P*-value	Multivariate analysis HR (95% CI)	P-value
Age (years)		< 0.001		< 0.001
< 60 years	1.000	–	1.000	
≥ 60 years	1.274 (1.112–1.461)	–	1.341 (1.169–1.538)	
Gender		0.850		–
Male	1.000	–	–	
Female	0.987 (0.861–1.131)	–	–	
Tumor location		0.140		–
Upper/Middle	1.000	–	–	
Lower	1.117 (0.964–1.294)	–	–	
Depth of invasion		< 0.001		< 0.001
pT1	1.000	–	1.000	–
pT2	1.402 (1.051–1.869)	0.022	1.085 (0.796–1.479)	0.606
pT3	2.404 (1.845–3.133)	< 0.001	1.557 (1.137–2.131)	0.006
pT4	3.437 (2.168–4.451)	< 0.001	2.721 (1.527–4.848)	0.001
Lymph node metastasis		< 0.001		0.016
pN0	1.000	–	1.000	–
pN1	1.725 (1.493–1.994)	< 0.001	1.354 (1.066–1.718)	0.013
pN2	2.241 (1.881–2.670)	< 0.001	1.530 (1.142–2.048)	0.004
Stage		< 0.001		0.036
I	1.000	–	1.000	–
II	1.973 (1.456–2.674)	< 0.001	1.432 (1.007–2.035)	0.046
III	3.657 (2.712–4.933)	< 0.001	1.705 (1.078–2.696)	0.023
Differentiation		0.122		0.046
G1	1.000	–		–
G2	1.411 (1.105–1.961)	0.040	–	–
G3	1.374 (0.984–1.918)	0.062	–	–
NLR		< 0.001		< 0.001
< 3.29	1.000		1.000	–
≥ 3.29	1.859 (1.678–2.060)		1.578 (1.373–1.814)	–
NMR		0.202		–
< 12.77	1.000			–
≥ 12.77	2.010 (1.716–2.355)		–	–
LMR		< 0.001		0.001
≥ 2.95	1.000	–	1.000	–
< 2.95	1.373 (1.205–1.566)	–	1.265 (1.107–1.445)	
RDW		0.049		0.366
< 15.05%	1.000		1.000	–
≥ 15.05%	1.1411.001–1.301)		1.063 (0.931–1.213)	–
PDW		0.016		0.104
< 13.65%	1.000	–	1.000	–
≥ 13.65%	1.280 (1.048–1.562)	–	1.181 (0.966–1.444)	–

### Survival analysis results of significance detection indicators

**Fig. 2:**
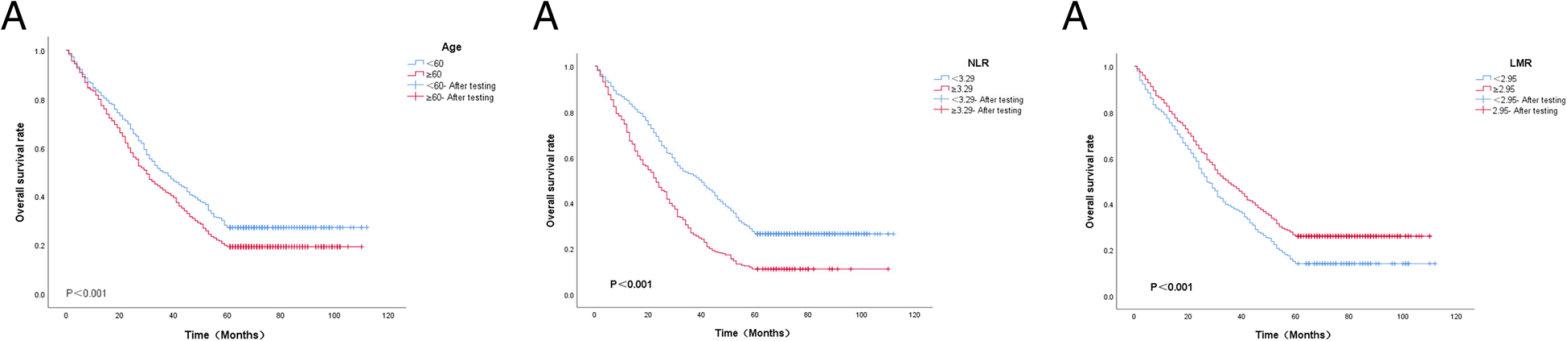
Kaplan Meier survival curve for the indicators of significance. NOTE: NLR:neutrophil to lymphocyte ratio;LMR:lymphocyte to monocyte ratio

Based on the Cox regression multivariate analysis, we selected Age, NLR, and LMR to establish the survival curve. From the (Fig. 2A-C), it showed that the age <  60, NLR < 3.52 and LMR ≥ 2.95 groups had higher overall survival.

### Nomogram development and internal validation

**Fig. 3:**
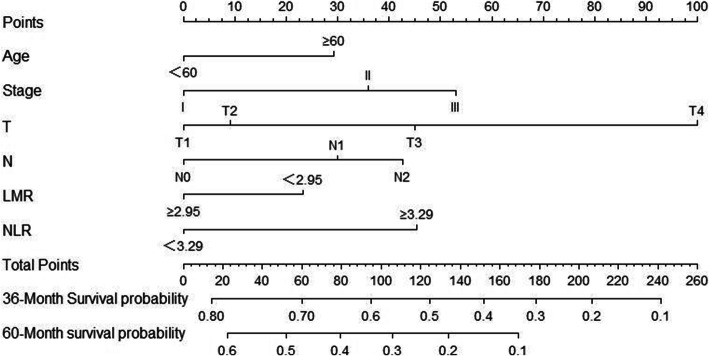
Evaluation of nomogram of CBC index in the patients with esophageal squamous cell cancer after esophagectomy. NOTE: To use the nomogram, the value attributed to an individual patient is located on each variable axis, and a line is drawn upwards to determine the number of points received for each variable value. The sum of these numbers is located on the total points axis, and a line is drawn downward to the survival axis to determine the likelihood of 3- or 5-year survival. NLR:neutrophil to lymphocyte ratio;LMR:lymphocyte to monocyte ratio

**Fig. 4:**
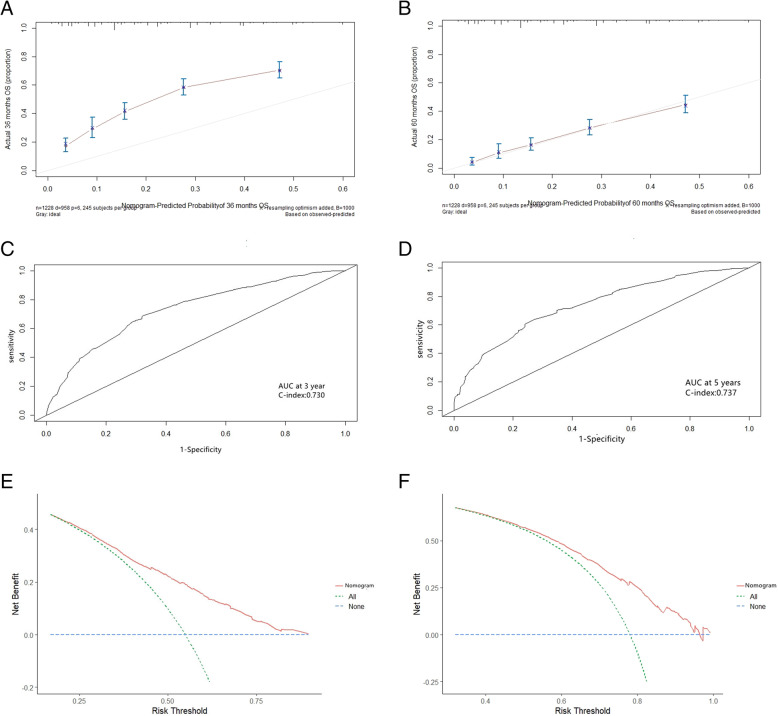
Performance validation for predicting 3-year and 5-year survival in the training cohort. NOTE: Calibration curve by nomogram, Pathological stage for 3-year (A) and 5-year (B) OS in the training cohort, Time-dependent receiver operating characteristic (ROC) curves by nomogram, Pathological stage for 3-year (C) and 5-year (D) OS in the training cohort. Decision curve analyses by nomogram, Pathological stage for 3-year (E) and 5-year (F) OS in the training cohort

Based on Cox regression multivariate analysis of the training cohort; the nomogram for predicting overall survival at 3 and 5 years in the training cohort was established by age, NLR, LMR, depth of invasion, lymph node metastasis, and stage (Fig. 3). The 3-year survival probability calibration plot indicated that the nomogram predicted risk overestimate the actual risk,the 5-year survival probability calibration plot indicated good accuracy between nomogram prediction and actual observation. Their C-index scores were 0.730 and 0.737, indicating their good discrimination. The decision curve showed that it had good potential for clinical application (Fig. 4A-F).

**Fig. 5:**
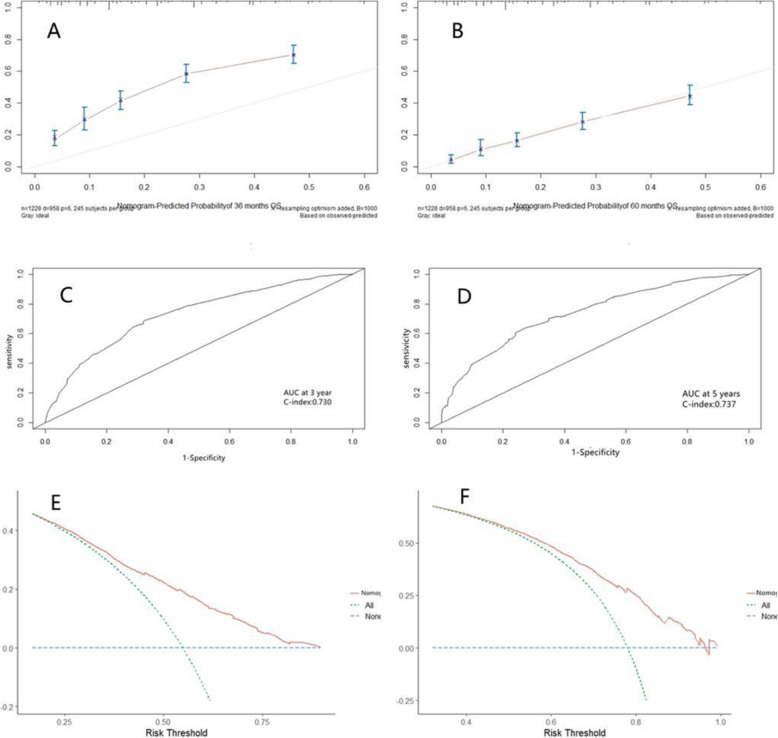
Performance validation for predicting 3-year and 5-year survival in the validation cohort. NOTE: Calibration curve by nomogram, Pathological stage for 3-year (A) and 5-year (B) OS in the validation cohort, Time-dependent receiver operating characteristic (ROC) curves by nomogram, Pathological stage for 3-year (C) and 5-year (D) OS in the validation cohort. Decision curve analyses by nomogram, Pathological stage for 3-year (E) and 5-year (F) OS in the validation cohort

At the end of the internal validation, the overall survival of every patient in the validation cohort was evaluated by a nomogram designed based on the training cohort. The calibration curves show good agreement in the 5-year survival probability between actual observed and nomogram predictions, and their 3-year actual observed risk was still overestimated. In the validation cohort, the 3- and 5-year C-index was 0.733 and 0.745. These results indicate that the Nobel plot is a more accurate and useful tool for predicting overall survival in patients with operable ESCC (Fig. 5A-F).

## Discussion

The incidence of esophageal cancer is high in the world. About 580,000 new cases were diagnosed worldwide, meanwhile, about 510,000 people died of esophageal cancer every year. In China, nearly 300,000 patients with esophageal cancer died in every year, accounting for more than 50% of the global deaths of esophageal cancer [8]. Now it is generally recognized that inflammation plays an increasingly important role in the development of tumors, and its mechanism may be include the inflammation releases cytokines and up-regulation of transcription factors, then leading to the generation and accumulation of a large number of oxygen free radicals, which could cause DNA damage and breakage in parenchymal cells, including stem cells, overexpression of proto-oncogenes, loss of function of tumor suppressor genes and up-regulation of genes that promote the cell cycle, leading to abnormal cell proliferation, thereby interfering with the stability of the body’s microenvironment, accelerating tumor growth, invasion, metastasis and other processes, affecting the prognosis of tumors [9, 10]. Many reports have shown that LMR, NLR and NMR, PDW, RDW as inflammatory indicators, were closely related to the prognosis of a variety of diseases and could be used as prognostic factors in a variety of malignant tumors [7, 11, 12]. In recent years, nomograms have been constructed for many cancers, and some of them were more reliable than traditional staging prediction [13]. Although the nomograms have many advantages, there were few nomograms to construct for ESCC by CBC. We established nomograms by CBC indicators and pathological parameters to predict the prognosis of ESCC and validate its model performance.

In this study, we investigated the effect of inflammatory indicators in blood routine on the prognosis of preoperative esophageal squamous cell carcinoma. Based on univariate Cox regression analysis of training cohort, NLR ≥ 3.29, LMR < 2.95, RDW ≥ 15.05% and PDW ≥ 13.65% were risk factors for preoperative esophageal squamous cell carcinoma prognosis; multivariate Cox regression analysis model showed that NLR ≥ 3.29 and LMR < 2.95 were independent risk factors for preoperative esophageal squamous cell carcinoma, which were the same as most studies; the HR of NLR ≥ 3.29 was 1.578 (1.373–1.814; *P* <  0.001) than NLR < 3.29, which was consistent with Arigami T [14] and Gao GD [15] studies, and NLR > 3.0 and NLR > 2.83 were effective predictors for preoperative esophageal carcinoma; the HR of LMR < 2.95 was 1.265 (1.107–1.445; P <  0.001) than LMR ≥ 2.95, Huang Y [16] et al. believed that preoperative LMR < 2.93 predicted poor prognosis of patients with esophageal squamous cell carcinoma. Hu G [17] et al. did a meta-analysis and also concluded that low LMR could predict poor prognosis of esophageal cancer, meanwhile, Li KJ [18] believed that LMR and NLR could be used to predict the survival rate of patients with advanced esophageal cancer who also received chemoradiotherapy, and confirmed that LMR and NLR could predict the survival rate of patients with preoperative esophageal cancer in this study. Among other related blood routine test indicators, PDW had distinct conclusions, Kawakita Y [19] et al. believed that PDW < 12.5% could predict poor prognosis of esophageal cancer, but in contrast to Song Q [20] et al., he believed that high PDW could predict poor prognosis of esophageal cancer, and in this study, PDW, RDW, and NMR were not independent prognostic factors of preoperative esophageal squamous cell carcinoma (*p* > 0.05), which may be different from the cut-off values of PDW, RDW, and NMR in these studies. The criteria and methods for determining cut-off values were diferent in diferent institutions; a suitable cut-off value cannot be proposed by statistical analysis. This may affect the results and lead to an inevitable potential bias, which may limit the value of NLR, LMR, RDW, PDW in clinical practice and even lead to distinct conclusions, therefore, defining NLR, LMR, RDW, PDW requires a standard, uniform cutoff. Among the pathological parameters, we concluded that pathological stage, depth of invasion, and lymph node metastasis were independent prognostic factors for preoperative esophageal squamous cell carcinoma, and differentiation was not an independent prognostic factor, which may be associated with most of the esophageal cancers being advanced. In this study, we tried to establish a nomogram based on blood routine and pathological parameters to predict the prognosis of ESCC. In the performance validation, its 3-year calibration curve indicated that its predictive probability overestimate the actual probability. 5-year calibration curve indicated that its predictive probability was consistent with the actual probability, which may be related to the low overall survival rate of ESCC and needs to be further explored; the decision curve analysis showed the potential of clinical application of the prediction model. This was also verified in the validation cohort. We believe that our model is a simple and easy tool for both physicians and patients to estimate the survival rate of esophageal cancer patients in the postoperative situation.

Several limitations should be acknowledged in the current study. First, the current study is a retrospective study, although it is a large sample, but other potential diseases affecting inflammatory cannot be completely ruled out. Secondly, excessive uncontrollable factors for laboratory test results of blood cell count-related indicators and excessive variation of indicators, which lead to its limited role in the judgment of clinical prognosis. Therefore, this study aimed at the expression level of peripheral blood cells in patients with esophageal cancer, and explored the relationship between a variety of inflammatory indicators and the prognosis of esophageal cancer, in order to provide new ideas for the prognosis of esophageal cancer. So we also expected to have a more scientific and rigorous prospective, multicenter study to verify our result.

## Data Availability

The datasets generated and/or analysed during the current study are not publicly available due requirements of Cancer hospital Affliated to Xinjiang Medical University, but it are available from the corresponding author on reasonable request
